# Supplementary material for: Machine Learning Model to Stratify the Risk of Lymph Node Metastasis for Early Gastric Cancer: A Single-Center Cohort Study
Source: Cancers (Basel). 2022 Feb 22;14(5):1121. doi: 10.3390/cancers14051121 (PMC8909118; doi:10.3390/cancers14051121)
Supplement: Supplementary file 1 [file cancers-14-01121-s001.zip › cancers-1576945-supplementary.pdf]

cancers

MDPI

Supplementary Materials

Machine Learning Model to Stratify the Risk of Lymph Node Metastasis for Early Gastric Cancer: A Single-Center Cohort Study

Ji-Eun Na [1,2,†], Yeong-Chan Lee [3,†], Tae-Jun Kim [1,*], Hyuk Lee [1,*], Hong-Hee Won [3], Yang-Won Min [1], Byung-Hoon Min [1], Jun-Haeng Lee [1], Poong-Lyul Rhee [1] and Jae J. Kim [1]

[1] Department of Medicine, Samsung Medical Center, Sungkyunkwan University School of Medicine, Seoul 06351, Korea; jieun90.na@samsung.com (J.-E.N.); yangwon.min@samsung.com (Y.-W.M.); lamsu.min@samsung.com (B.-H.M.); jh2145.lee@samsung.com (J.-H.L.); pl.rhee@samsung.com (P.-L.R.); jaej.kim@samsung.com (J.J.K.)

[2] Department of Medicine, Inje University Haeundae Paik Hospital, Busan 48108, Korea

[3] Department of Digital Health, Samsung Advanced Institute for Health Sciences & Technology (SAIHST), Sungkyunkwan University of Medicine, Seoul 06351, Korea; conan_8th@naver.com (Y.-C.L.); wonhh@skku.edu (H.-H.W.)

* Correspondence: taejunk91@gmail.com (T.-J.K.); leehyuk@skku.edu (H.L.); Tel.: +82-234-103-409 (T.-J.K. & H.L.); Fax: +82-234-106-983 (T.-J.K. & H.L.)

† These authors contributed equally to this work.

Table S1. Best hyperparameters selected from the search algorithm.

	Logistic regression	RF	SVM
Best hyperparameters	{'C': 0.0164, 'penalty': 'l2'}	{'C': 1.0437, 'gamma': 0.0108}	{'n_estimators': 500, 'max_depth': 10, 'criterion': 'entropy'}
Hyperparameter candidates	{'penalty': ['l1', 'l2', 'elasticnet', 'none'], 'C': loguniform(1e-2, 1e2)}	{'C': loguniform(1e0, 1e3), 'gamma': loguniform(1e-2, 1e2)}	{'n_estimators': [100, 200, 300, 500, 700, 1000], 'criterion': ['gini', 'entropy'], 'max_depth': [10, 20, None]}

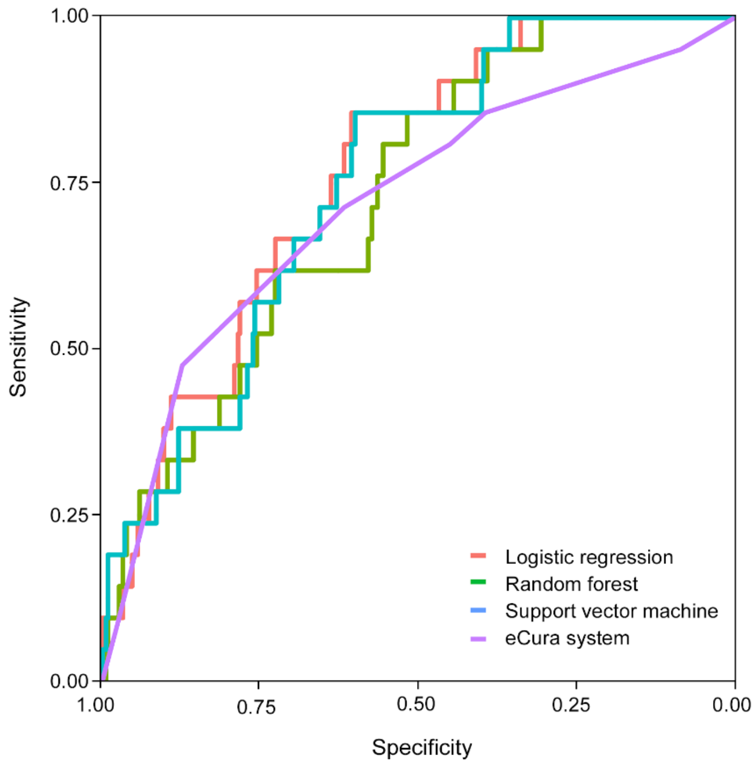

Prediction model	AUROC (95% CI)	NRI (95% CI)	Specificity (%) at high-sensitivity cutoff 95%
Logistic regression	0.76 (0.67-0.85)	0.46 (0.31–0.62)	39
Random forest	0.73 (0.63-0.83)	0.04 (-0.11–0.20)	38
Support vector machine	0.75 (0.65-0.83)	0.21 (0.16–0.25)	38
eCura system	0.72 (0.58-0.83)		9

Figure S1. Performance of the machine learning model and eCura system for predicting lymph node metastasis in patients with non-curative resection after endoscopic resection.
